# Surgical Treatment for Simple Isolated and Displaced Olecranon Fractures: Comparison between Plate and Tension Band Wire Fixation

**DOI:** 10.3390/jcm13061815

**Published:** 2024-03-21

**Authors:** Serena Maria Chiara Giardina, Gianluca Testa, Enrica Rosalia Cuffaro, Mirko Domenico Castiglione, Marco Sapienza, Alessia Caldaci, Pierluigi Cosentino, Angelo Raffa, Vito Pavone

**Affiliations:** Department of General Surgery and Medical Surgical Specialties, Section of Orthopaedics and Traumatology, A.O.U.P. Policlinico Rodolico–San Marco, University of Catania, 95123 Catania, Italy; serenamc.giardina@gmail.com (S.M.C.G.); enricacuffaro@outlook.it (E.R.C.); mirkodomenicocastiglione@gmail.com (M.D.C.); marcosapienza09@yahoo.it (M.S.); alessia.c.92@hotmail.it (A.C.); pierluigi-cosentino@hotmail.it (P.C.); angelo57raffa@gmail.com (A.R.); vitopavone@hotmail.com (V.P.)

**Keywords:** olecranon, tension-band wiring, plate, fracture, clinical-functional outcome, range of motion, complications, reoperation

## Abstract

**Background**: Olecranon fractures are common injuries of the upper limb in adults. Simple displaced trasverse fractures are generally surgically treated with tension-band wiring (TBW) or plate fixation (PF). The purpose of this retrospective study is to compare the clinical-functional outcome, complications and reoperation rates between TBW and PF for Mayo IIA fractures. **Methods**: 72 patients treated with PF or TBW at our institution, completed our survey and clinical evaluation and their demographic and clinical data were recorded and analysed. The clinical-functional outcomes were evaluated assessing ROMs and three validated scoring systems: the Disabilities of the Arm, Shoulder, and Hand (DASH), the Mayo Elbow Performance Score (MEPS) and the Patient American Shoulder and Elbow Surgeons Standardized Elbow Assessment score (pASES-e). **Results**: 38 patients (53%) underwent TBW and 34 (47%) PF. The mean DASH, MEPS and pASES-e scores were respectively 14.5 ± 17.2, 80.5 ± 14.7 and 83.6 ± 12.4 in the TBW group and 21 ± 21.7, 75.6 ± 15.3 and 75.1 ± 19.2 in the PF group (*p* = 0.16, *p* = 0.17 and *p* = 0.03). The mean duration of surgery and hospitalisation period were longer in the PF group (*p* = 0.002, *p* = 0.37) whereas the complication and reoperation rates were higher after TBW (*p* = 0.15, *p* = 0.24). **Conclusions**: According to the literature, both TBW and PF resulted comparable valid surgical options for the treatment of simple isolated displaced olecranon fractures. Our results corroborate previous findings, showing good/excellent outcomes without significant differences.

## 1. Introduction

Olecranon fractures are common adult injuries and account for 10% of the upper limb fractures [1,2] They represent the most frequent elbow fractures. The overall incidence is estimated at 11.5–12 per 100,000 population [1,3]. Because of its subcutaneous location, olecranon fractures may occur as a result of direct trauma, indirect trauma, or a combination of both [4]. These fractures commonly show a bimodal distribution: they occur after high-energy trauma in young patients and after simple falls in older patients due to poor bone quality [5]. These fractures are all intraarticular injuries, and they benefit from anatomic reduction and restoration of the joint surface to allow early range of motion and restore functional elbow motion and strength. Olecranon fractures range from simple nondisplaced fractures to complex fracture-dislocations. Approximately 85% of all olecranon fractures in the adult population can be described as simple isolated and displaced transverse fractures.

Multiple classification systems for olecranon fractures exist (Mayo, AO, Colton, Schatzker, Horne and Tanzer), but none of them is unequivocally accepted nor can be used in clinical setting to provide direct and reliable guidance on strategies to be followed [6]. One of the most common is the Mayo classification where simple isolated and displaced fractures are described as Mayo IIA.

These fractures are traditionally managed operatively, and several methods of open reduction and internal fixation used. The chosen method of surgical procedure usually depends on the amount of bone loss, the amount of comminution, the ability to reduce the articular surface, and whether the fracture is defined as stable or unstable [7,8]. Two main methods of operative management of olecranon fractures are used: tension-band wiring (TBW) and plate fixation (PF). Others, such as intramedullary fixation and excision of the proximal fragment with triceps advancement, are rare.

Historically, TBW was recommended for the treatment of simple isolated and displaced fractures, whereas plate fixation (PF) is reserved for more complex patterns [9,10].

Tension-band wiring is a simple, approved, and low-cost technique. It is the most widely used method. However, its disadvantages include the high number of symptomatic prominences of Kirschner wires (K-wires) and the subsequent removal of metalwork [11,12,13]. Plate fixation is indeed better and provides superior fracture reduction in all fracture patterns as well as resistance against screw pull-out in osteoporotic bone [14,15]. Thus, the use of plates has become more common in recent years but there is still controversy regarding the optimal surgical treatment for simple isolated, displaced fractures.

Several studies have compared outcomes and complications between TBW and PF, but no statistical differences were reported.

The purpose was to verify whether a treatment choice was superior in the management of this specific olecranon fracture pattern. Our zero hypothesis was that no difference would emerge between the two surgical options studied either in terms of outcomes and post-treatment complication and removal rates.

## 2. Materials and Methods

This was a retrospective medical record review on all patients surgically treated with open reduction internal fixation (ORIF) for an olecranon fracture at the Unit of Orthopedics and Traumatology of the University Hospital Policlinico “G. Rodolico-San Marco” in Catania in a period between January 2017 and June 2022. We identified all the patients who had suffered a simple isolated, displaced fracture of the olecranon and had been treated either with tension-band wire or plate fixation.

In the period under analysis, a total of 115 patients with a Mayo IIA–AO/OTA 2U1B1(d) olecranon fracture were treated surgically with tension-band wiring or plate fixation; 25 were excluded from the study because they met exclusion criteria or did not meet inclusion criteria; 18 were lost to follow-up because they were not available to participate or could not be reached using the contact details provided. Finally, 72 patients were eligible and successfully completed the survey.

The inclusion criteria were: (1) simple isolated and displaced fracture; (2) ORIF treatment with either single plate or tension-band wiring; (3) greater than 16 years of age at the time of surgery; and (4) minimum one-year period follow-up. The exclusion criteria were: (1) complex fracture pattern (comminuted fracture; associated elbow fractures with attention to radial head, coronoid process or distal humeral fracture; fracture-dislocation; open fracture); (2) additional ipsilateral upper extremity injury; (3) pathological fracture; (4) greater than 85 years of age at the time of surgery; (5) ORIF technique other than TBW and single plate fixation such as double plates or intramedullary screw or nail; (6) follow-up less than one year; and (7) incomplete documentation or radiographs.

All patients’ demographic and clinical data, such as limb involved, complications, reoperations, and hospitalization time were collected and reviewed from medical records, discharge sheets, and ambulatory reports. Using a picture archiving and communication system (PACs), pre-operative anteroposterior and lateral injury X-rays were carefully reviewed in order to classify fracture pattern and concomitant injuries as well as CT scans in bone windowing, when available. The two most common classification systems were applied: the Mayo classification and the latest version of the classification proposed by AO/OTA. Thus, all Mayo IIA and AO/OTA 2U1B1(d) fractures were identified for being included into the study.

Operative reports were also reviewed to determine the implants, the surgical technique utilized in each case and the duration of the surgery. All patients underwent TBW or plate fixation performed by orthopaedic surgeons working at our Unit, both orthopaedic consultants and orthopaedic residents working alongside them.

### 2.1. Surgical Technique

All operations were performed with the patients under general or regional anesthesia (axillary block) with sedation and in supine decubitus position. A longitudinal posterior approach was adequate for this fracture pattern. The fracture was exposed through a posterior midline incision with the proximal end curving to the lateral aspect of the olecranon.

The tension-band wiring (TBW) technique was first introduced by Weber and Vasey in 1963 [16,17]. More recently, it was modified and formalized by the AO group becoming the gold standard procedure for the treatment of intra-articular simple transverse olecranon fractures [18]. The working principle of TBW is to convert tensile forces applied across the fracture by the longitudinal pull of the extensor mechanism into a compressive dynamic force at the joint surface which hold the fracture site more closely leading to better fracture healing [12,19,20,21,22].

After a preliminary reduction, two parallel 1.6-mm Kirschner wires were placed antegrade across the fracture site through the proximal end of the olecranon as perpendicular to the fracture line as possible. Some surgeons prefer to angle the K-wires volarly to penetrate and engage the distal anterior cortex, whereas others place them parallel to the long axis of the ulna in the intramedullary canal.

Once the proximal end of the K-wires is bent 180°, the fibers of the triceps tendon should be split sharply with a scalpel at the site of the wires to allow to cut and bent ends to be impacted against the cortex. These gaps between the fibres of the triceps will then be closed over the wires to cover them and reduce protruding and prevent backing out.

An 18-gauge stainless steel wire is passed through a pre-drilled hole perpendicular to the ulnar shaft made with a 2-mm drill in the distal fragment, approximately 3.5–4 cm away from the fracture site line and 5 mm away from the posterior cortex. The wire is then passed in a figure-of-eight configuration and crossed over the posterior aspect of the fracture to the insertion of the triceps brachii muscle at the olecranon. Both wire ends are then united with a twist and tightened with tongs. To produce symmetric tension at the fracture site and more rigid fixation, two twisted knots [19] are placed one radial ad one ulnar (usually 3 or 4 twists are needed to achieve the appropriate tension). These are later bent down against the cortex in order not to irritate the soft-tissues. When the figure-eight wire loop is tensioned, the tension band mechanism starts working.

Plate fixation (PF) has been used mainly for the fixation of comminuted olecranon fractures in which tension band wire fixation is not feasible. Other possible indications are complex fracture patterns requiring a high stability fixation: coexisting coronoid fractures, oblique fractures distal to the midpoint of the trochlear notch, and Monteggia fracture-dislocations with associated olecranon fractures [6].

The PF adapts to the shape of the proximal ulna, lying as close as possible to the bony surface of the olecranon to ensure greater reliability and stability of the fixatio.

There are different types of plates that can be used: One-third tubular, 3.5 mm contoured limited contact dynamic compression (LC–DCP), 3.5 mm reconstruction, hook plates, and pre-contoured locking plates are frequently used [23]. The choice of a specific plate and fixation technique is determined by the fracture pattern, the quality of the bone and the surgeon’s preference.

Plates are commonly applied posteriorly along the dorsal surface of the ulna and contoured around the tip of the olecranon. This represents the side of the olecranon where the tension is greatest and thus makes the structure biomechanically more capable of resisting the bending forces of the triceps tendon [24,25]. Fragments are reduced directly with the help of small pointed reduction forceps and can be temporarily held with k-wires to the proximal and distal ulnar between each other or the trochlea of the distal humerus.

The plate may be applied once the reduction is obtained. If an anatomic plate is not used, then the plate must be contoured to fit the proximal ulna bending around the tip of the olecranon and following its curvature. Proximally, the plate may sit off the triceps insertion [26] or a longitudinal incision can be made to allow the device to sit flush with the posterior cortex. The fixation is then completed by applying locking screws to provide rigidity. AO suggests the plate should be anchored to the bone, whenever possible, with three screws proximal and three screws distal to the fracture applied bi-cortically and without protruding into the joint [18].

After surgery, patients followed an identical protocol for TBW and plate which provided elbow immobilization for two weeks with the use of a splint. Careful passive and active mobilization were then removed followed by active movement against resistance under physiotherapy guidance at four weeks. After hospital discharge, patients were followed-up regularly: For the first month, they were monitored weekly for wound healing and removal of stitches and splint. Patients were then seen at 1 month, 3–6 months, and 1 year postoperatively for clinical and radiological follow-up. The radiographic outcome was assessed for fracture union, but also for possible complications such as hardware migration/failure, malunion/nonunion, and post-traumatic osteoarthritis.

### 2.2. Clinical and Radiographic Evaluation

After hospital discharge, patients were followed-up regularly following a protocol identical for TBW and plate. For the first month, patients were monitored weekly for wound healing and removal of stitches and plaster. Patients were then seen at 1 month, 3–6 months, and 1 year postoperatively for clinical and radiological follow-up. The radiographic outcome was assessed for fracture union, but also for possible complications such as hardware migration/failure, malunion/nonunion. And post-traumatic osteoarthritis.

Patients were divided into two groups according to the type of treatment they received (TBW or PF) and were evaluated in terms of age, sex, involved side, clinical-functional outcomes, hospital stay, surgical duration, complications, and reoperation. All data were maintained in a de-identified database.

To assess the functional outcome after surgical therapy of olecranon fractures, we measured the elbow joint ROMs and use three different validated scoring systems described in the current literature: the Disabilities of the Arm, Shoulder and Hand score (DASH), the Mayo Elbow Performance Score (MEPS), and the Patient American Shoulder and Elbow Surgeons Standardized Elbow Assessment score (pASES-e) [27,28]. The aim of the scoring systems is to simplify complex clinical situations and to ensure reproducibility of the results, make comparisons, and extrapolate findings.

The DASH is one of the most common tools utilized to evaluate the daily function following injuries of the upper limb (Attachment A1 in Appendix A) [29,30]. It is a 30-item questionnaire in which patients are asked about the degree of difficulty in performing activities of daily living and specific symptoms such as pain, weakness, or paraesthesia of the upper limb. Shoulder, arm, and hand are assessed as a functional unit whose degree of impairment is measured via score. All items of DASH are scored with a five-point scale: 1 = no difficulty; 2 = mild difficulty; 3 = moderate difficulty; 4 = severe difficulty; and 5 = unable. Scores range from 0 (no disability) to 100 (most severe disability).

The Mayo Elbow Performance Score, or MEPS, was developed specifically for assessing elbow function following fractures (Attachment A2 in Appendix A) [31,32,33]. The MEPS measures elbow function across four subscales: pain (45 points), ulnohumeral stability (10 points), range of motion (20 points), and five daily functional tasks (25 points). The score ranges from 5 to 100 points and is summarized into four outcome categories. Scores are weighted markedly in favor of the subjective parameters such as pain and daily function. The outcome can be rated as poor (less than 60 points), fair (60–74 points), good (75–89 points), or excellent (90–100 points).

The American Shoulder and Elbow Surgeons-Elbow, or ASES-e, is a standardized elbow assessment tool proposed by the Research Committee of the American Shoulder and Elbow Surgeons (ASES) (Attachment A3 in Appendix A). ASES-e is suitable for assessing elbow function regardless of the underlying pathological condition. It consists of two sections: a patient self-evaluation questionnaire (patient ASES-e: pASES-e) and a form intended for the physician to record the degree of clinically detectable elbow impairment (clinical ASES-e: cASES-e) [34,35]. The pASES-e form is further subdivided into three sections: pain, function, and satisfaction. In the first section, the pain evaluation is based on a visual analogical scale (from 0 = no pain, to 10 = worst pain ever). The second section contains 12 questions concerning the function of right and left arms, and answers are rated on a four-point scale (from 0 = unable to do, to 3 = no difficult). The maximum score for the function of each arm is 36 with lower scores indicating worse function. In the third section the patient is asked to rate his/her satisfaction with the surgery on a scale of 0 to 10. The pASES-e total score results from the sum of the pain and function subscales equally weighted and can thus range from 0 to 100. The pain score was derived by subtracting the resultant pain score from 50. The function score was derived with the following formula: 50/3 × (arithmetic mean of the 12 function items).

The experimental data collection phase of the study took place at two different points in time. At first, the information necessary to determine the scores was recorded by telephone consultation. Patients were then contacted via telephone, verbally consented for participation in the study, and were asked to provide the required answers. Subsequently, the patients were clinically evaluated in our department for ROMs measurements. The text was translated into Italian, and validated versions of the questionnaires were administered. Orthopedic goniometers were used to measure the joint’s ranges of motion.

### 2.3. Statistical Analysis

Data collected were analyzed and statistically elaborated using Microsoft Excel 365 for Windows (Microsoft, Redmond, WA, USA). Characteristics of the study patients were described as mean values (with standard deviation) or numbers (with percentages) as appropriate. Student’s *t*-test was used to detect any significant differences in continuous variables such as age, functional outcomes, surgery time, hospital stay, and time elapsed before reoperation between the two groups. In case of normal distribution independent samples, a *t*-test was used to define the two-sided probability of statistical significance and in the analyses that reported a F-test, a *p*-value less than 0.05 (variances of the two samples cannot be assumed to be equal) were applied; *t*-tests were corrected for unequal variances (Welch test).

A Chi-squared test was used in categorical variables to determine whether there was a statistically significant difference between the expected frequencies and the observed ones. It was used to analyze statistical differences for sex, complications, and reoperation rates between the two groups. Values of *p* < 0.05 were accepted as statistically significant.

## 3. Results

### 3.1. Patient Demographics

The average patient age was 46.1 ± 21.7 years (range 16 to 80 years). The cohort was composed of 46 males (64%) and 26 females (36%). Male mean age was 40.3 ± 20.8 years (range 16 to 78 years), whereas female mean age was 56.2 ± 19.7 years (range 16 to 80 years). There were 40 fractures (56%) involved the left elbow, and 32 (44%) in the right elbow.

There were 38 patients (53%) treated with TBW and the remaining 34 (47%) were treated with PF. The average patient age in the TBW group was 43.9 ± 22.5 years compared with 48.4 ± 20.9 years in the PF group (*p* = 0.38). There were 26 males (68%) and 12 females (32%) within the TBW group and 20 males (59%) and 14 females (41%) in the PF group with no difference in composition between cohorts (*p* = 0.39).

### 3.2. Hospitalization and Surgery Duration

Medical records showed that the mean time of hospital stay was 9.3 ± 3.7 days (range 3–18 days) (Table 1). The mean hospitalization period was 8.6 ± 4 days in the TBW group (range 4–18 days) and 9.5 ± 3.2 days in the PF group (range 3–15 days). There was no evidence of statistical significance in the duration of hospitalization between the two groups (*p* = 0.37). We calculated the duration of each procedure based on the start and end time of surgery recorded on the operative notes.

In both groups, surgery was performed with the same patient positioning, type of anesthesia and surgical approach. The mean surgery time for both groups was 117.9 ± 59.1 min (range 40–260) (Table 1). The mean duration of surgery was 86.6 ± 30.9 min in the TBW group (range 40–165) and 143.3 ± 61.3 in the PF group (range 60–260). The duration of surgery was significantly higher in the PF group (*p* = 0.002).

### 3.3. Patient Outcomes

**Figure 1:**
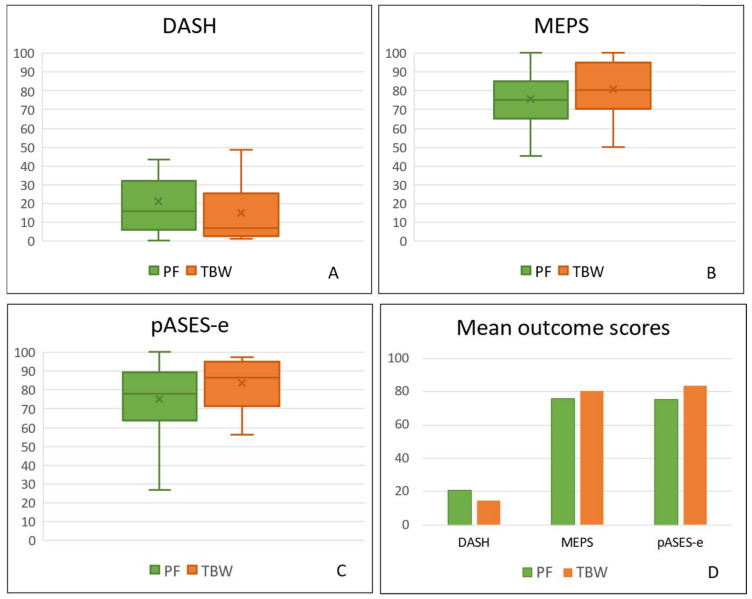
Outcome analysis between TBW and PF group. (**A**) DASH Score; (**B**) MEPS; (**C**) pASES-e; (**D**) whole outcome scores comparison.

For all the patients enrolled in the study, the mean DASH score was 17.6 ± 19.6 with a range 0–84.2. The mean MEPS was good at 78.2 ± 15.1 with a range of 45–100. The mean pASES-e score was 79.6 ± 16.4 with a range 26.7–100. Among the TBW group, patients had a mean DASH score of 14.5 ± 17.2 with a range 0.8–62.5. The mean MEPS was good—80.5 ± 14.7 with a range 50–100. The mean pASES-e score was 83.6 ± 12.4 with a range 55.8–97.2. In PF, the mean DASH score was 21 ± 21.7 with a range 0–84.2. The mean MEPS was good—75.6 ± 15.3 with a range 45–100. The mean pASES-e score was 75.1 ± 19.2 with a range 26.7–100. Comparing the score systems used, the TBW group showed slightly better values for all the outcomes. The DASH score was lower (14.5 ± 17.2 < 21 ± 21.7), MEPS was higher (80.5 ± 147 > 75.6 ± 15.3), and pASES-e score was higher (83.6 ± 12.4 > 75.1 ± 19.2). However, not all these differences were found to be statistically significant as shown in Figure 1. There was no difference between the two groups in terms of the DASH (*p* = 0.16) and MEPS (*p* = 0.17), whereas pASES-e was significant (*p* = 0.03) (Table 2).

The values of joint ROM at a mean of one year after radiographic union resulted to be comparable as shown in Table 3. In the TBW group, patients had a mean of 138.02 ± 4.92° of flexion and 5.26 ± 22.3° of extension. The mean pronation reached was good at 67.89 ± 6.20°; the mean supination was 82.97 ± 5.60°. In the PF group, the mean flexion-extension was 138.2 ± 2.34° and 4.57 ± 21.13°. The mean pronation was 68.00 ± 6.52°, and the mean supination was 83.25 ± 5.02°. There was no statistically significant difference between the two groups in terms of the flexion (*p* = 0.34), extension (*p* = 0.26), pronation (*p* = 0.43), or supination (*p* = 0.30).

### 3.4. Surgical Complications

Post-operative complications were reported in 11 of the 72 patients (15.3%). Eight complications occurred in the patients who underwent TBW fixation (21.1%). These included six cases of metalwork irritation (15.8%), one case of nonunion (2.6%), and one case of implant mobilization (2.6%). In the plate fixation cohort, there were three postoperative complications (8.8%). These included one case of metalwork irritation (2.9%), one case of ulnar neuropathy (2.9%), and one case of infection (2.9%). There was no statistically significant difference between the incidence of eight complications in the TBW cohort versus three in the PF group (*p* = 0.15) (Table 4).

Clinical notes were examined to determine the rate of reoperation and the reason for intervention in each case. There were 10 patients who required further surgery (13.9%). In the TBW group, seven patients needed reoperation (18.4%). Six of these underwent elective removal of metalwork for ongoing implant irritation (15.8%), and one (2.6%) had his TBW construct revised to plate because of a nonunion fixation accompanied by bone autograft. Three patients were treated with plate fixation and underwent reoperation for plate removal (8.8%). One of them (2.9%) needed also an ulnar neurolysis. Even for the rate of reoperation there was no statistically significant difference between the TBW group versus the PF group (*p* = 0.24).

The average time elapsed between the ORIF surgery and the reoperation was 12.4 ± 5.7 months (range 4–20 months). In the TBW group the second operation took place on average after 11.4 ± 6.2 months (range 4–20 months), whereas in the PF group this was after 14 ± 4 months (range 9–20 months). No statistically significant difference was found between the two cohorts (*p* = 0.57) (Table 5).

## 4. Discussion

In this study, we evaluated the clinical-functional outcomes of a retrospective series of simple isolated, displaced olecranon fractures treated with TBW and PF with minimum 1-year follow-up. Several studies in the literature have already tried to compare these two surgical techniques to identify which would give better results. Classically, TBW fixation has been considered the most appropriate treatment for simple displaced olecranon fracture, whereas PF has been reserved for more comminuted fractures [9,14,19,36,37].

However, in current clinical practice, TBW has been questioned as the gold standard technique, and PF has become more common even for more simple fracture patterns. The study of Hutchison et al. [38] failed to prove the theory that posterior tensile forces are converted to compressive force at the articular fracture site. Brink et al. [39] showed that the tension band principle only works under certain circumstances, which are usually not met in daily life, thus suggesting the presence of a static component acting on the fracture in order to keep reduction and achieve bone healing. Schneider et al. [40] debunked the popular belief that tension band wire fixation is a surgical technique easy to learn and to apply, drawing attention to potential risks, errors, and complications.

On the other hand, Wilson et al. [41] compared compression at the fracture site during rest and simulated muscle activity in a cadaveric model. They showed that, in comparison with tension bands in the treatment of transverse olecranon fracture, plate fixation can secure greater compression both over the entire fracture and specifically on the articular surface involved.

Many previous articles have often reported similar clinical outcomes for TBW and PF, but the results remain mixed with data supporting both fixation methods. A Cochrane systematic review of 2014 [42] of six randomized controlled trials and 244 surgically-managed olecranon fractures showed that there is no sufficient evidence for robust conclusions on the effects of different surgical treatment options. This meta-analysis concluded that more randomized blinded clinical studies are needed to determine the optimal surgical management of simple isolated fractures.

A more recent systematic review from 2021 [43] analyzed 229 patients from five studies and found no differences both in clinical and patient-rated outcomes between displaced olecranon fractures treated with the two most common fixation techniques (TBW and PF). There was insufficient evidence to draw robust conclusions on the clinical superiority of one treatment over another.

In 1992, Hume and Wiss [44] were among the first to evaluated in a randomized trial the results of PF versus TBW in different olecranon fractures patterns. They found better clinical outcome and lower complication rate in the PF cohort. No validated patient-reported outcome measures were used, but symptomatic metalwork was seen more frequently in the TBW group (42%) than in the PF group (5%).

Duckworth et al. [45] more recently reported an ambitious prospective and randomized trial including adult patients with an isolated and displaced fracture of the olecranon. The aim of the study was to determine if any difference existed between TBW and PF with respect to the outcome. The data demonstrated that both techniques provided comparable outcomes for DASH score, MEPS and joint ROM. The overall complication rate was higher following TBW fixation (63.3% TBW vs. 37.5% PF) because of an increased rate of symptomatic implant removal (50% TBW vs. 21.9% PF).

Tarallo et al. [46] compared TBW fixation and PF for both Mayo 2A and 2B fractures. No significant differences in functional and clinical outcome were observed between the two groups. Specifically, type 2A fractures had slightly better values for all the outcomes evaluated for the PF group, except for flexion and pronation, but without any significant differences. Likewise, there was no significative difference in complications and hardware removal although rates were higher in TBW than the plate as a whole. However, a statistically significant increase in hardware removal was seen in the TBW group only when grouping both 2A and 2B fracture patterns.

Delsole et al. [47] compared TBW fixation constructs versus the hook plate, and they both used Mayo-type 1, 2, and 3 fractures (the largest subgroup contained Mayo 2A fractures). Good outcomes were achieved in both groups according to MEPS and ranges of motion. No significant differences were reported except for flexion, which was worse in the PF group than in the TBW one. There was also a non-significant increased rate of symptomatic hardware (30.4% vs. 20%) and removal (9% vs. 0%) in TBW group.

Claessen et al. [48] worked on a large retrospective study involving olecranon fractures treated with plate or tension-band wire for the purpose of predicting reoperation and implant removal. Their analysis grouped both Mayo 2A and 2B fractures and a variety of different implants and showed a reoperation rate of 25%. When comparing reoperation and implant removal rates, no significative difference was shown between fracture types and TBW versus plating (22% vs. 26% respectively). However, there was a statistically significance increase in both reoperation and request for reoperation in younger patients and those of female sex. Schliemann et al. [49] compared the clinical and radiological outcomes in TBW fixation constructs versus locking compression plates looking at Mayo 2A fractures in isolation. The outcomes were good with no difference between the cohorts, but twice as many patients required TBW removal as plate removal.

Powell et al. [50] compared patients treated surgically with either TBW or locking PF for Mayo 2A fractures of the olecranon. In this case, patient outcomes were calculated using the QuickDASH, but no statistically significant difference were found when comparing the two cohorts. In terms of post-surgical complications and reoperation, significantly higher rates were shown in TBW group: 39.6% and 33.3%, respectively.

Gathen et al. [51] compared the outcomes of patients treated with PF or TBW after isolated olecranon fracture. Different fracture patterns were evaluated, but Mayo 2A were the most represented (52%). Both treatment groups showed good to excellent outcomes regardless of which scoring system was used including DASH. There were no significant differences in complication rates (32.5% in all cases) even if symptomatic metalwork was more common in the TBW group (40% TBW vs. 25% PF). The mean duration of surgery and the mean time of hospital stay were compared as well with longer timing in the in the PF group.

Recent studies focused their attention on complication and re-operation rates between TBW and locking PF for olecranon fractures. Rantalaiho et al. [52] published a ten-year retrospective analysis, although identifying higher rates of complications (50%) and re-interventions (40%) recorded no statistically significant difference in the rate of early complications (49% vs. 62%) or reoperations (38% vs. 53%) between patients treated with TBW and PF even when only looking at Mayo 2 fractures.

Oputa et al. [53] published a multi-center study and found that the overall complication rate was 25%, and the overall re-operation rate was 17%. There were no significant differences between the two procedures, although complication rates were 28% and 22% and re-operation rates of 15% and 19% for PF vs. TBW, respectively.

Previous studies include an interesting comparative economic analysis between the two surgical approaches [45,49,50]. They argue that although plate implant has higher costs per se, the higher frequency of complications and implant removal makes the overall costs of TBW are comparable if not higher.

In our cohort, we found that both fixation strategies yielded similar results. We used not only three different patient-reported outcome scores (DASH, MEPS, pASES-e) but also the evaluation of elbow range of motion. The findings indicated that both with TBW and PF excellent/good clinical outcomes can be obtained.

Although the absolute data deriving from the scoring systems used would seem to suggest slightly better results in TBW, clinical-functional outcomes resulted in statistically comparable values with a significative difference just for pASES-e. This seamlessly mirrors the existing literature: although several authors [46,51], using only one or two of the scoring systems we used, achieved slightly better values overall, in no case was one technique better than the other.

Indeed, our results are similar to the literature with regard to the time of hospital stay and the duration of surgery, although for these elements have fewer studies with which to compare. For example, the time frames proposed by Gathen et al. [51] were both slightly longer than ours, but still with extended times for patients treated with plate fixation.

Our findings showed a complication rate of 15.3% in all patients, although the rate was higher in the TBW group at 21.1% compared to 8.8% in the PF. Painful hardware irritation requiring hardware removal was the most common complication after open reduction and internal fixation for olecranon fractures. The higher incidence of metalwork irritation, as well as the higher rate of hardware removal, were reported after TBW versus plating (15.8% vs. 2.9% and 18.4% vs. 8.8%, respectively). Our results are concordant with other findings in the literature even if showed significantly lower rates: our statistics are far away from the 50% described by Rantalaiho et al. [43] or the 32.5% by Gathen et al. [51]. In our cohort, as in the literature, TBW was more problematic in terms of complication and subsequential removal, but our numbers are lower than expected.

This apparent discrepancy may be due to several reasons. It is not always easy to assess and quantify hardware-related pain after surgery leading to removal as multiple subjective and objective variables are involved.

Several studies argued that this could be due to the prominence of the tip of the K-wires as a result of their proximal migration out of the proximal end of the ulna. Pain or irritation could, therefore, be affected not only by the subjective threshold of tolerance, but also by the thickness of the overlying soft tissue or by the poor consistency of the patient’s bone, which does not guarantee an adequate grip for the K-wires to resist against the traction of the triceps and maintain their position.

To confirm this, Edwards et al. [54] reported that many patients had their implant removed for the most varied reasons, not always objectifiable. But at the same time, they reported that many others renounced the hardware removal for the fear of another surgery. Long-term follow-up studies showed rates of complications and subsequential hardware removal of >80% [3,36,55]. In addition to the studies mentioned above, Macko and Szabo [56] reported painful hardware following TBW in 75% of patients, with hardware removed in 65% of all patients. Villanueva et al. [13] reported a 46% of hardware removal at a mean four-years of follow-up. In Terstappen at al. [57], implant removal rates were 84% at a mean 7-years follow-up; in 79% of cases this was due to wire prominence.

Longer follow-up may allow us to record more numerous post-treatment severities and elective removals later. Therefore, our follow-up periods may not be extended enough to conclude for certain that there are or are not statistically significant differences between complications and reoperation rates.

Furthermore, as discussed, Edwards et al. [54] reported that 78% of patients for removal of tension-band wire or plate referred to a different surgeon than the one who had originally performed the surgery, especially if a long time has passed. This showed that removal rates may be much higher than believed and recorded by surgeons and institutions. If this is the case, then even our study may significantly underestimate the hardware removal rates for both types of surgeries.

One of the main limitations of our study is its retrospective design. Different surgeons performed the procedures over the study period and, even if case record analysis revealed that similar surgical techniques were employed, a bias may be represented by each surgeon’s personal device preference or expertise.

It was not always easy to contact patients because of incomplete or incorrect contact details and poor patient compliance. Another limitation is represented by the follow-up length. As mentioned, a longer period of study may have allowed a different identification and quantifications of complications and reoperations. Finally, the COVID-19 pandemic led to restrictions and fear that could have delayed participation. Many patients were lost on follow-up, and others confessed having renounced seeing their surgeons despite having post-treatment discomforts.

## 5. Conclusions

We conclude that open reduction and internal fixation obtained using tension-band wire or plate achieved similarly good-to-excellent results for simple isolated/displaced olecranon fractures. Both the results of the clinical-functional outcomes and the complications and implant removal rates proved that both techniques represent an effective surgical option for this fracture pattern.

Although we found that TBW was superior to PF with respect of the clinical-functional outcome, complications and hardware removal were most frequently observed just after TBW. These differences in findings between the two surgical treatment groups did not reach statistical significance.

No clear algorithm exists to determine which technique is more appropriate in our specific scenario, and the choice between these two surgical treatments remains controversial. Both procedures can result technically difficult to perform needing a not easy learning curve, and success depends on multiple various factors not only construct-related but also patient and surgeon-related. However, we believe that patients should be carefully counselled and advised about the pros and cons of both techniques considering the particular cases and individual surgeon’s preferences. Further studies with a larger sample size, longer follow-up, and higher methodological standards, e.g., prospective, randomized controlled trials, are needed to address the issue.

## Figures and Tables

**Table 1:** Comparison of hospitalization and surgery duration between TBW and PF group.

	Hospitalization (days)		Surgery Duration (min)	
	Mean ± 1 SD	Range	Mean ± 1 SD	Range
TBW group	8.6 ± 4.0	3–18	86.6 ± 30.9	40–165
PF group	9.5 ± 3.2	4–18	143.3 ± 61.3	60–260
*p* value	0.37		0.002	
Overall population	9.3 ± 3.7	3–18	117.9 ± 59.1	40–260

**Table 2:** Comparison of patient clinical outcomes between TBW and PF group.

	DASH		MEPS		pASES-e	
	Mean ± 1 SD	Range	Mean ± 1 SD	Range	Mean ± 1 SD	Range
TBW group	14.5 ±17.2	0–22	80.5 ± 4.7	50–100	83.6 ± 12.4	55.8–97.2
PF group	21.0 ± 21.7	0–84.2	75.6 ± 5.3	45–100	75.1 ± 19.2	26.7–100
*p* value	0.16		0.17		0.003	
Overall population	17.6 ± 9.6	0–84.2	78.2 ± 5.1	45–100	79.6 ± 16.4	26.7–100

**Table 3:** Comparison of range of motion between TBW and PF group.

	Range of Motion (°)			
	Flexion	Extension	Pronation	Supination
	Mean ± 1 SD	Mean ± 1 SD	Mean ± 1 SD	Mean ± 1 SD
TBW group	138.02 ± 4.92	5.26 ± 22.3	67.89 ± 6.20	82.97 ± 5.60
PF group	138.2 ± 2.34	4.57 ± 21.13	68 ± 6.52	83.25 ± 5.02
*p* value	0.34	0.26	0.43	0.3
Overall population	138.10 ± 3.75	4.93 ± 21.75	67.94 ± 6.35	83.10 ± 5.32

**Table 4:** Complications analysis e comparison between TBW and PF group.

	Metalwork Irritation	Nonunion	Implant Migration	Infection	Ulnar Neuropathy	Total Complications
	N (%)	N (%)	N (%)	N (%)	N (%)	N (%)
TBW group	6 (15.8)	1 (2.6)	1 (2.6)	0 (0)	0 (0)	8 (21.2)
PF group	1 (2.9)	0 (0)	0 (0)	1 (2.9)	1 (2.9)	3 (8.8)
*p* value						0.15
Overall population	7 (9.8)	1 (1.4)	1 (1.4)	1 (1.4)	1 (1.4)	11 (15.3)

**Table 5:** Comparison reoperation rates and time elapsed after ORIF surgery between TBW and PF group.

	Reoperations	Time Interval (months)	
	N (%)	Mean ± 1 SD	Range
TBW group	7 (18.4)	11.4 ± 6.2	4–20
PF group	3 (8.8)	14 ± 4.0	9–20
*p* value	0.24	0.57	
Overall population	10 (13.9)	12.4 ± 5.7	4–20

## Data Availability

Data is contained within the article.

## Appendix A

- Attachment A1

DASH—Disability Arm Shoulder Hand

- Attachment A2

MEPS—Mayo Elbow Performance Score

- Attachment A3

pASES-e—Patient American Shoulder and Elbow Surgeons—Elbow
